# 5-Azacytidine Enhances the Radiosensitivity of CNE2 and SUNE1 Cells In Vitro and In Vivo Possibly by Altering DNA Methylation

**DOI:** 10.1371/journal.pone.0093273

**Published:** 2014-04-01

**Authors:** Wei Jiang, Ying-Qin Li, Na Liu, Ying Sun, Qing-Mei He, Ning Jiang, Ya-Fei Xu, Lei Chen, Jun Ma

**Affiliations:** State Key Laboratory of Oncology in South China, Sun Yat-sen University Cancer Center, Guangzhou, People’s Republic of China; Gustave Roussy, France

## Abstract

The radioresistance of tumor cells remains a major cause of treatment failure in nasopharyngeal carcinoma (NPC). Recently, several reports have highlighted the importance of epigenetic changes in radiation-induced responses. Here, we investigated whether the demethylating agent 5-azacytidine (5-azaC) enhances the radiosensitivity of NPC cells. The NPC cell lines CNE2 and SUNE1 were treated with 1 μmol/L 5-azaC for 24 h before irradiation (IR); clonogenic survival was then assessed. Tumor growth was investigated in a mouse xenograft model *in vivo*. The apoptosis, cell cycle progression and DNA damage repair were examined using flow cytometry, immunofluorescent staining and western blotting. Promoter methylation and the expression of four genes epigenetically silenced during the development of NPC were evaluated by pyrosequencing and real-time PCR. We found that pretreatment with 5-azaC significantly decreased clonogenic survival after IR compared to IR alone; the sensitivity-enhancement ratio of 5-azaC was 1.4 and 1.2 for CNE2 and SUNE1 cells, respectively. The combined administration of 5-azaC and IR significantly inhibited tumor growth in the mouse xenograft model, and enhanced radiation-induced apoptosis *in vitro* compared to 5-azaC alone or IR alone. 5-AzaC also decreased promoter methylation and upregulated the expression of genes which are epigenetically silenced both *in vitro* and *in vivo* in NPC. Thus, 5-azaC enhance the radiosensitivity of both the CNE2 and SUNE1 cell lines, possibly by altering DNA methylation levels and increasing the ability of irradiated cells to undergo apoptosis. The use of 5-azaC combined with IR maybe represent an attractive strategy for the treatment of NPC.

## Introduction

Nasopharyngeal carcinoma (NPC) is prevalent in southeastern Asia, especially in southern China where the incidence is approximately 25-50 per 100,000 population per year [1]. Radiotherapy is the primary treatment modality for locally- and regionally-confined NPC. Despite recent significant advances in the treatment of NPC, local recurrence is frequently observed [2]. Radiation resistance is one of the major obstacles that leads to locoregional recurrence of NPC during treatment [3].Therefore, the identification of effective radiosensitizing agents to enhance the radiosensitivity of NPC cells may help to decrease both tumor recurrence and radiation-associated morbidity.

More recently, increasing evidence supports the suggestion that genome-wide changes in methylation levels are associated with the radiosensitivity of cancer cells [4], [5], [6]. Epigenetic modifications, specifically DNA hypermethylation that leads to the aberrant silencing of multiple tumor suppressor genes, are believed to play a pivotal role in variety of cellular events [7], [8], including alterations in apoptosis, cell cycle progression, mitotic checkpoint regulation and DNA repair; all of these mechanisms have been considered to mediate radiosensitizing effects [4], [9].

DNA hypermethylation has been frequently reported in NPC [10], [11]. Aberrant promoter methylation of tumor suppressor genes, such as Ras association domain family member 1A (*RASSF1A*), Cyclin-dependent kinase inhibitor 2A (*CDKN2A*), Reprimo (*RPRM*) and Stratifin (*14-3-3σ*), have been commonly detected in NPC [11], [12], [13]. These genes play important roles in cell cycle control, apoptosis, checkpoint activation, and translational regulation [14], [15], [16], [17]. With the increasing awareness of epigenetic abnormalities in NPC, counteracting these changes using methyltransferase inhibitors such as 5-azacytidine (5-azaC) [18] may be a potential strategy of radiosensitizing NPC cells.

The relationship between DNA methylation changes and radiosensitivity in NPC, however, remain unknown. This study aimed to investigate the radiosensitizing effect and possible mechanism of the demethylating agent 5-azaC in NPC cells *in vitro* and *in vivo*. Our data demonstrated that 5-azaC enhances the level of cellular radiosensitivity and was associated with increased rates of apoptosis, potentially as a consequence of the altered methylation levels.

## Methods and Materials

### Cell culture and treatment

The human NPC cell lines CNE2 and SUNE1 were obtained from the Cancer Center of Sun Yat-sen University. The cells were maintained in RPMI-1640 (Gibco, USA) containing 10% fetal bovine serum, 100 U/ml penicillin, and 100 μg/ml streptomycin at 37°C in 5% CO_2_. Cells were exposed to 5-azaC (Sigma, USA) and/or irradiated at 12.7 Gy/min at room temperature using a RS 2000 X-ray Biological Irradiator operated at 160 kV/50 mA (Rad Source Technologies, USA). The treatment groups were as follows: control group (PBS); 5-azaC group (0 to 5 μmol/l); ionizing radiation (IR) group (0 to 8 Gy irradiation); and combined treatment group (pretreatment with 5-azaC for 24 h followed by IR). The 5-azaC was dissolved in phosphate-buffered saline (PBS); control cells were treated with media containing the same volume of PBS.

### Cell proliferation assay

CNE2 or SUNE1 cells were seeded at 2000 cells/well in 96-well plates and 24 h later, the cells were treated with 5-azaC (0, 50, 100, 500, 1000, 3000, or 5000 nmol/l). Cell proliferation was measured using the 3-(4, 5-dimethylthiazol-2-yl)-2, 5- diphenyl-tetrazolium bromide (MTT; Sigma, USA) assay after 24, 48 and 72 h of 5-azaC treatment. The absorbance of the converted dye was measured at 490 nm using a microplate reader (Bio-Tek ELX800, USA).

### Colony formation assay

Single cells were seeded and treated with 5-azaC (0 or 1 μmol/l) alone, IR (0, 2, 4, 6 or 8 Gy) alone, or 5-azaC and IR. After culture for 10-14 days, the cells were fixed in ice-cold methanol, stained with Giemsa solution and colonies containing >50 cells were counted. Cell survival curves were fitted using the linear-quadratic (LQ) formula: surviving fraction (SF)  =  exp (-αD-βD^2^) [19], wherein α and β are the radiobiological cell survival parameters within the treatment volume, and D is irradiation dose; the sensitizer enhancement ratio (SER) was determined from the survival curves of irradiation in the presence and absence of the tested compounds using the following the equation: SER  =  D_0_ untreated cells/D_0_ treated cells, where D_0_ values represent the radiation dose that led to 37% cell survival [20].

### In vivo tumor xenograft model

Twenty 4-week-old female BALB/c nu/nu nude mice were purchased from the Guangdong Experimental Animal Center. All protocols were approved by the Institutional Animal Care and Use Committee of Sun Yat-Sen University (IACUC SYSU, NO.10212100E). CNE2 cells (1×10^6^) were subcutaneously injected into the left hind flank region. After the xenograft tumors reached 0.5 cm diameter, the mice were randomly assigned to four groups (n = 5) and treated as follows: the control group received twice weekly intraperitoneal (i.p.) injections of 100 μl PBS; the 5-azaC group received twice weekly i.p. injections of 100 μl 5-azaC (4 mg/kg); the IR group received twice weekly i.p. injections of 100 μl PBS and xenograft irradiation (8 Gy, once) at second week; and the 5-azaC + IR group received twice weekly i.p. injection of 100 μl 5-azaC (4 mg/kg) and xenograft irradiation (8 Gy, once) at second week. Tumor sizes and body weights were measured weekly. Tumor volumes (TVs) were calculated using the formula TV  =  LD^2^/2 (where L was the longest diameter and D was the shortest diameter) [21].

### Cell cycle and apoptosis analysis

Cells were seeded in 6-well plates, incubated for 24 h, and treated with PBS, 5-azaC (1 μmol/l), IR (6 Gy), or 5-azaC + IR as described above. Cell cycle progression and apoptosis were analyzed with the Cell Cycle and Apoptosis Kit (Keygentec, China) using a CytomicsTM FC500 flow cytometer and CXP analysis software (Beckman Coulter, USA) following the manufacturer’s instructions. Cell cycle analysis was performed using CXP analysis software; apoptotic cells were considered to include cells stained Annexin V (+)/propidium iodide (PI) (−) (lower right quadrant, early apoptosis) and late cells stained Annexin V (+)/PI (+) (upper right quadrant, late apoptosis) [22].For each sample, at least 10,000 cells were analyzed.

### Immunofluorescent staining

Immunofluorescent staining of phosphorylated histone H2AX (γH2AX) was performed as previously described [23]. Cells were cultured on a chamber slide and treated with IR (6 Gy), or 5-azaC + IR as previously described. Cells then were fixed in 4% paraformaldehyde, permeabilized in Triton X-100 for 15 min, incubated with anti-γ-H2AX antibody (Cell Signaling Technology, USA) overnight at 4°C, then incubated with secondary antibodies (Cell Signaling Technology, USA) for 1 h at 37°C. Nuclei were counterstained using 4′, 6′-diamidino-2-phenylindole (DAPI). The number of γH2AX-positive foci was determined in at least 50 cells at 400× magnification using a fluorescent microscope (Olympus); γH2AX repair kinetics were determined at 0, 1, 6, 24 and 48 h after irradiation.

### RNA isolation and quantitative real-time RT-PCR

Total RNA was isolated using TRIzol reagent (Life Technologies, USA); cDNA was synthesized from 1 μg total RNA using the RevertAid First Strand cDNA Synthesis Kit (Thermo Scientific, USA). Real-time RT-PCR was performed on a Bio-Rad CFX96 sequence detection system (Bio-Rad Laboratories Inc., USA) using Platinum SYBR Green qPCR SuperMix-UDG reagents (Life Technologies, USA). All reactions were incubated at 95°C for 3 min, followed by 40 cycles at 95°C for 15 s, annealing at 60°C for 15 s, and elongation at 72°C for 7 min. Primer sequences were obtained from previously published data for *RASSF1A, CDNK2A, RPRM, 14-3-3σ*, and glyceraldehyde-3-phosphate dehydrogenase (*GAPDH*) [24], [25], [26], [27], [28]. Glyceraldehyde-3-phosphate dehydrogenase (*GAPDH*) was amplified as an endogenous control, and relative expression levels were calculated using the 2^−ΔΔCT^ method [29].

### Western blot analysis

Equal amounts of protein were separated by 10% SDS-polyacrylamide gels SDS-PAGE (Bio-Rad) and transferred to polyvinylidene difluoride (PVDF) membranes (Millipore, USA). Membranes were blocked with blocking buffer (5% non-fat milk powder, 0.1% Tween 20 in TBS) for 1 h, followed by incubation with rabbit polyclonal antibodies in blocking solution overnight at 4°C. The membranes were then washed three times in TBST and incubated with horseradish peroxidase (HRP)-conjugated secondary antibody (1∶5000 dilution) for 1 h. After three final washes, the blots were visualized using an ECL detection system (Abcam, USA). Primary antibodies and concentrations used are indicated as follows: anti-DNA-PK (1∶1000; Cell Signaling Technology, USA), anti-phospho-S2056 DNA-PKcs (1∶1000; Cell Signaling Technology), anti-XRCC4 (1∶500; Proteintech, China). anti-XLF (1∶500; Proteintech), anti-RPA2 (1∶500; Proteintech), and anti-RAD51 (1∶500; Proteintech).

### DNA extraction and bisulfite pyrosequencing methylation analysis

Genomic DNA was isolated from cells treated with 1 μmol/l 5-azaC for 3 days using the EZ1 DNA tissue Kit (Qiagen, Germany). Bisulfite modification was performed using the EpiTect Plus DNA Bisulfite Kit (Qiagen) with 1 μg genomic DNA. Methylation of *RASSF1A, CDKN2A, RPRM*, and *14-3-*3σ in the bisulfite-treated DNA samples was quantitatively analyzed by bisulfite pyrosequencing as previously described [30], Pyrosequencing was carried out with primers designed by the PyroMark Assay Design Software 2.0 (Qiagen). Primer sequences and PCR conditions for bisulfite pyrosequencing are outlined in the Table 1. The sequencing reaction and quantitation of methylation was carried out using a PyroMark Q24 instrumentand software (Qiagen). Percentage methylation was calculated by averaging across all CpG sites interrogated.

**Table 1:** Sequences of the primers used for pyrosequencing methylation analysis and the cycling conditions.

Gene	Primers	Sequencing primer	No. of CpGs	Annealing temperature (°C)
*RASSF1A*	F: 5’-TAGTAAATAGGATTAGGAGGGTTAGGG-3’	5’-GTATAGTAAAGTTGGTTTTTAGA-3’	7	56
	R: 5’-biotin- CCTCCTTCCTCCCCTCCTCACA-3’			
*RPRM*	F: 5’-GGGTTAGAGGGGTGGGAAG-3’	5’-GTTGGAGGAATAGGTG-3’	6	55
	R: 5’-biotin- AACTCCCACCACCCAAAAACTTT-3’			
*CDKN2A*	F: 5’-GGAGGAAGAAAGAGGAGGGGT-3’	5’-GGGTTGGTTGGTTATTAGA-3’	7	56
	R: 5’-biotin- CAACCAATCAACCRAAAACTC-3’			
*14-3-3σ*	F: 5’-GAGTAGGGTTTTTTATTTGAAGATGAAGG-3’	5’-ATTTGAAGATGAAGGGT-3’	5	56
	R: 5’-biotin- TCTTACTAATATCCATAACCTCCTAAT-3’			

F, forward; R, reverse.

### Statistical analysis

Each *in vitro* experiment was repeated independently three times. Data is presented as the mean ± SD values. Statistical analysis was performed using SPSS version 13.0 (SPSS, Chicago, IL, USA). Differences between groups were compared with the Student’s *t*-test and one-way ANOVA was used for multiple comparisons. Two-tailed *p* values<0.05 were considered significant.

## Results

### Cytotoxicity of 5-azaC in NPC cells in vitro

**Figure 1:**
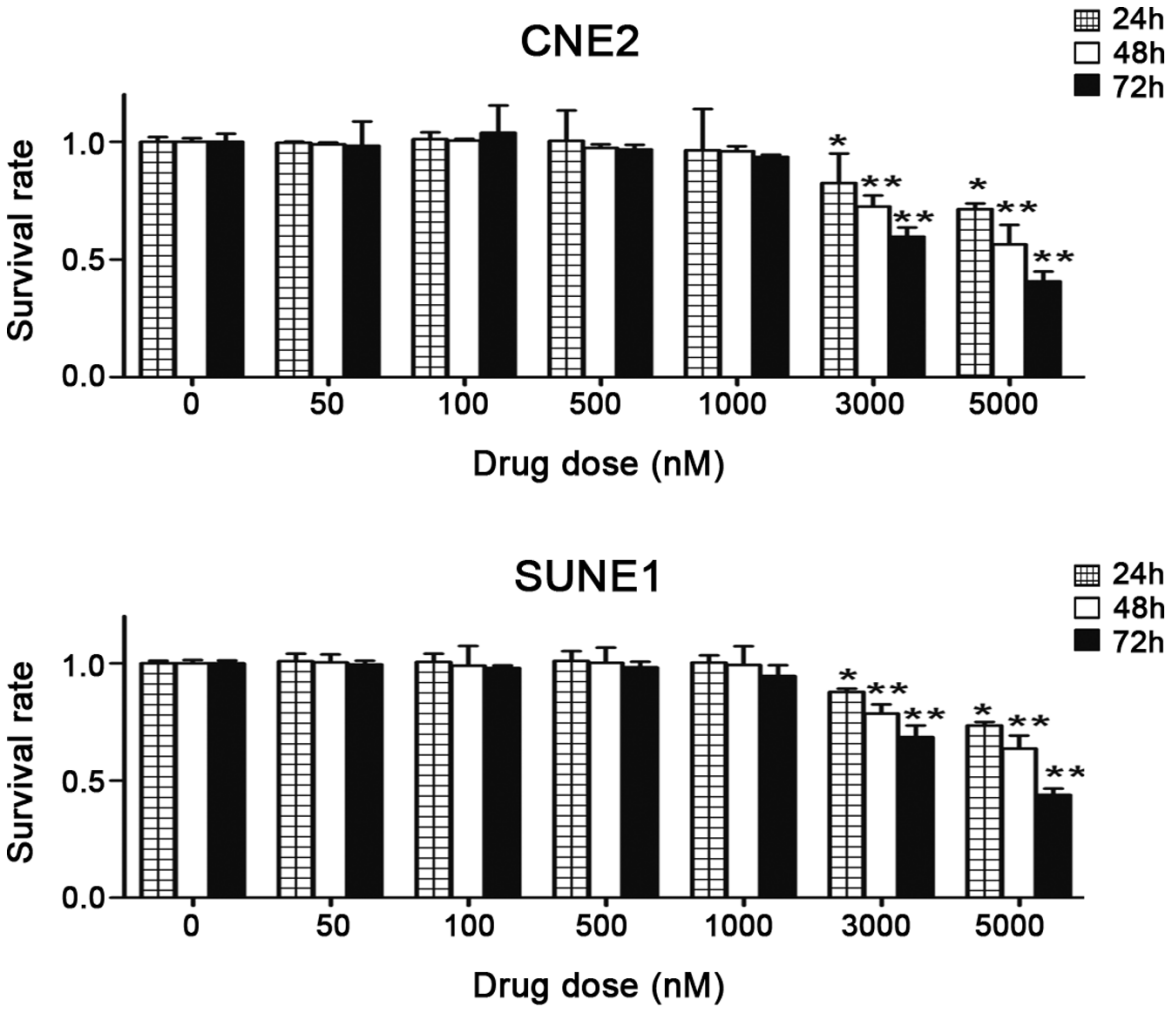
Effects of 5-azaC on the survival of NPC cells *in vitro*. As the drug concentration increased, the growth inhibition ratio for CNE2 and SUNE1 cells increased. After treatment with 3000 or 5000-azaC for 72 h, the survival rate was significantly reduced compared to the cells in the PBS control group (Student’s *t*-test ,*P<0.05; **P<0.01).

To investigate the cytotoxic effects of 5-azaC in NPC cells, CNE2 and SUNE1 cells were cultured with 0, 50, 100, 500, 1000, 3000, or 5000 nmol/l of 5-azaC. Cell proliferation was measured by the MTT assay after 24, 48, and 72 h of exposure to 5-azaC. Compared with the control group, no significant differences were observed in the survival rates of CNE2 and SUNE1 cells treated with 50 nmol/L to 1 μmol/L 5-azaC for 72 h (*p*>0.05). However, at 72 h, 3 μmol/L 5-azaC resulted in the cytotoxicity in CNE2 and SUNE1 cells (survival rate 60±3.86% and 68±4.94%, respectively; *p*<0.01), and major growth suppression was observed at concentrations of 5 μmol/L (survival rate 41± 4.24% and 44±2.68%, respectively; *p*<0.01) or higher (Fig. 1). These results demonstrate that concentrations of ≤ 1 μmol/L 5-azaC do not supress the survival or proliferation of NPC cells *in vitro*.

### 5-AzaC increases the radiosensitivity of NPC cells in vitro

**Figure 2:**
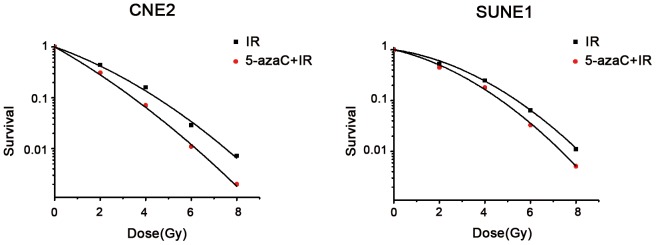
Effect of 5-azaC on the colony-forming ability of NPC cells *in vitro*. Clonogenic survival of CNE2 and SUNE1 cells irradiated with a single-dose of 0, 2, 4, 6, 8 Gy after pretreatment with 1 μmol/L (5-azaC +IR) or PBS (IR).

Based on the results above, we treated cells with 1 μmol/L 5-azaC to evaluate whether this demethylating agent increased the radiosensitivity measured in clonogenic survival assays. The survival rates of CNE2 and SUNE1 cells in the 5-azaC + IR group decreased significantly as the dose of radiation increased, compared to the IR alone groups (*p*<0.05) (Fig. 2).

The α and β parameters obtained using the L-Q model are displayed in Table 2. An increase of α or β has been suggested to represent an enhanced contribution due to the interaction of potentially lethal damage (PLD) or sublethal damage (SLD) [31], respectively. Increasing α and/or decreasing β values indicate higher radiosensitivity. When the 5-azaC treated and untreated groups were compared, the α value increased (CNE2 0.52±0.02 vs. 0.34±0.02, *p*<0.01; SUNE1 0.34±0.05 vs. 0.25±0.05, *p*<0.01) and the β value decreased (CNE2 0.032±0.003 vs. 0.034±0.01, *p* = 0.82; SUNE1 0.028±0.02 vs. 0.029±0.01, *p* = 0.95). SF2 indicates the survival rate after irradiation with 2 Gy; a higher SF2 indicates increased radioresistance. The SF2 values of two cells in the IR only groups were significantly higher than combined groups (CNE2 44%±0.02% vs. 31%±0.02%, SUNE1 54%±0.02% vs. 45%±0.02%, respectively; both *p*<0.01). The sensitivity-enhancement ratio (SER) of SF2 in CNE2 and SUNE1 cells was 1.4 and 1.2, respectively. Clonogenic assays showed that the α values and SER increased, while SF2 values decreased in the group pretreated with 5-AzaC before irradiation. These results show that pretreatment with 5-azaC increases the radiosensitivity of CNE2 and SUNE1 cells.

**Table 2:** Radiobiological parameters for CNE2 and SUNE1 cells treated with 1 μmol/L 5-azaC (mean ± SD, n = 3).

Cell line	Treatment condition	SF2	α	β
CNE2	Untreated	0.44±0.02	0.34±0.02	0.034±0.01
	5-azaC	0.31±0.02**	0.52±0.02**	0.032±0.003
SUNE1	Untreated	0.54±0.02	0.25±0.05	0.029±0.01
	5-azaC	0.45±0.02**	0.34±0.05**	0.028±0.02

α and β are experimentally derived parameters for LQ formula; SF2 indicates the survival rate after irradiation with 2 Gy. **p<0.01 (Student’s *t*-test , 5-azaC vs. untreated cells).

### 5-AzaC enhances the radiosensitivity of NPC cells in vivo

To confirm that the enhancement of radiation sensitization observed in vitro could be translated into an in vivo tumor model, a tumor growth delay assay was performed in mice using CNE2 cells grown subcutaneously (sc).We first compared the tolerability by examining the relative body weights and tumor volumes of xenografts throughout the study (Fig. S1). When the dose of 5-azaC ≤ 4 mg/kg was given twice weekly, body weights rose steadily (*p*>0.05; Fig. S1A–C) and tumor growth was not significantly suppressed (*p*>0.05; Fig. S1D, S1E) in each mouse group throughout the study.

**Figure 3:**
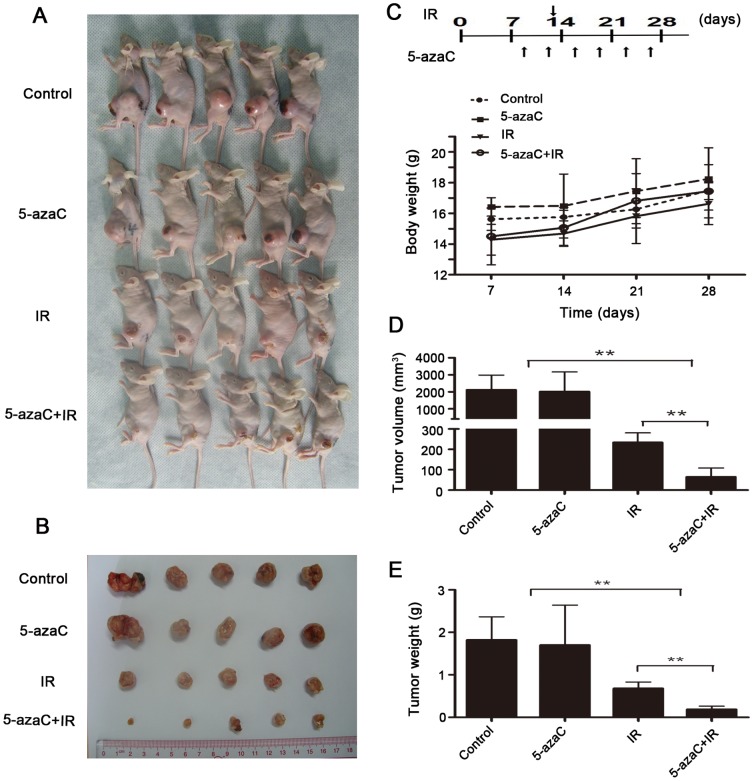
Effect of 5-azaC on the radiosensitivity of NPC *in vivo*. Mice bearing CNE2 tumor xenografts were randomized into four groups: control, 5-azaC, IR, or 5-azaC + IR (n = 5). (A, B) Images of the tumor bearing mice (A) and excised tumors (B). (C, upper part) The animals were treated and euthanized on day 28 of treatment. (C, lower part) Body weight of the mice during the study (one-way ANOVA, *p*>0.05). (D) The average tumor volume (mm^3^) for each group is shown (Student’s *t*-test, **p<0.01). (E) Average weight of the excised tumors (g) at the end of the study. (Student’s *t*-test, **P<0.01).

Accordingly, we examined the radiosensitizing effect of the twice weekly administration of 4 mg/kg 5-azaC in nude mice bearing xenograft tumors. During the observation period,, no significant differences were observed in the average weight of the mice in the four experimental groups (*p*>0.05; Fig. 3A–C). Tumor growth rates were not delayed in the control group treated with PBS and the experimental group treated with 5-azaC (*p*>0.05; Fig. 3A, 3B, 3D, 3E). However, significantly smaller tumor volumes (3.6-fold) and lower tumor weights (4.5-fold) were observed in the 5-azaC + IR group compared to the IR alone group (64.34%±44.02 mm^3^ vs. 233.99%±47.73 mm^3^, *p*<0.01; 0.19%±0.07 g vs. 0.68%±0.15 g, *p*<0.01; Fig. 3A, 3B, 3D, 3E). These results indicate that 5-azaC enhances *in vivo* radiosensitivity in the CNE2 xenograft model.

### 5-AzaC enhances radiation-induced apoptosis, but does not affect cell cycle progression and DNA double-strand breaks (DSBs) repair

**Figure 4:**
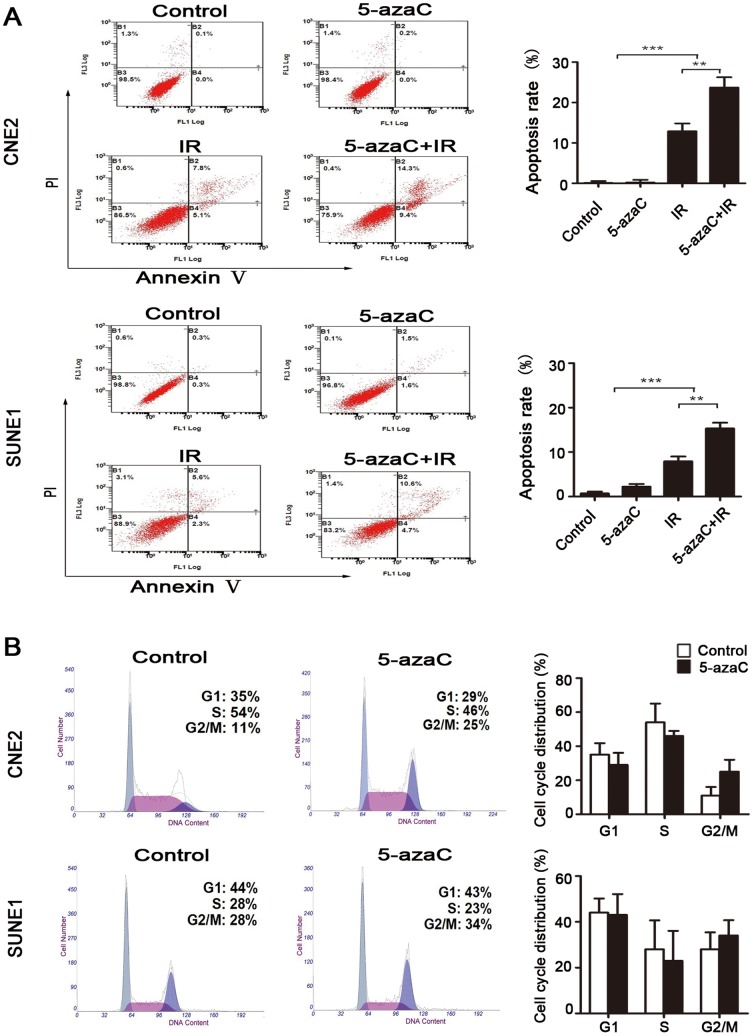
Effect of 5-azaC on the apoptosis and cell cycle in NPC cells *in vitro*. (A) CNE2 and SUNE1 cells were treated with 1 μmol/L 5-azaC and/or irradiated (IR) at 6 Gy. The rate of apoptosis (Annexin V (+)/PI (−) plus Annexin V (+)/PI (+)) was measured by flow cytometry at 72 hours after treatment (Student’s *t*-test, **P<0.01; ***P<0.001). (B) CNE2 and SUNE1 cells were treated with 5-azaC or PBS for 24 h and stained with PI to examine the cell cycle distribution by flow cytometry (Student’s *t*-test, *p*>0.05). Representative flow cytometric plots and quantification of the cell cycle distribution are shown.

To assess the possible mechanism underlying the enhanced radiosensitivity of cells treated with 5-azaC, we evaluated the apoptosis, the cell cycle progression and the expression and activation of regulators for radiation-induced DSBs in CNE2 and SUNE1 cells. Flow cytometry analysis indicated that there was no significant difference in the rate of apoptosis (Annexin V (+)/PI (−) plus Annexin V (+)/PI (+)) between the control and 5-azaC groups of either CNE2 or SUNE1 cells that did not receive IR (both *p*>0.05; Fig. 4A). IR alone resulted in 10.83%±1.92% of CNE2 cells and 8.97%±1.10% of SUNE1 cells undergoing apoptosis after 72 h of treatment. Importantly, 5-azaC increased the amount of IR-induced apoptosis to 21.30%±2.56% and 16.63%±1.30% in CNE2 and SUNE1 cells, respectively (both *p*<0.01; Fig. 4A).

Flow cytometry analysis showed that, when treated with and without 5-azaC, there was no statistical difference in the percentage of cells in the G2/M phase (CNE2 15.91%±5.13% vs. 27.43%±7.09%, *p* = 0.08; SUNE1 21.23%±7.44% vs. 32.74%±6.74%, *p* = 0.12) or G1 phase (CNE2 43.10%±6.80% vs. 30.27%±7.13%, *p* = 0.09; SUNE1 43.14%±6.20% vs. 32.39%±9.12%, *p* = 0.17) at 24 h in both cell lines(Fig. 4B).

**Figure 5:**
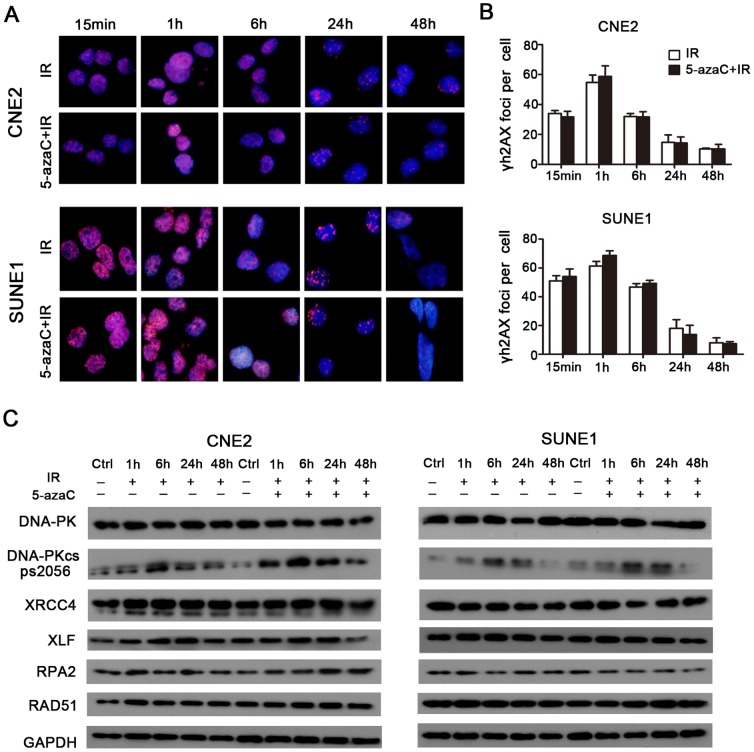
Effect of 5-azaC on DNA double strand break repair in NPC cells *in vitro*. CNE2 and SUNE1 cells were pretreated with 5-azaC, irradiated at 6Gy and subjected to γH2AX staining at the indicated times. (A) Images of γH2AX-positive foci (pink) and DAPI nuclear counterstaining (blue). (B) Double strand break repair kinetics (Student’s *t*-test, *p*>0.05). (C) Western blot assays of the expression levels of *DNA-PK, Phospho-DNA-PK (Ser2056), XRCC4, XLF, RPA2* and *RAD51* over time in CNE2 and SUNE1 cells after IR.

Immunofluorescent staining indicated that the number of γH2AX foci per cell that received combined 5-azaC/IR treatment was not significantly different over time compared to the corresponding IR only groups (*p*>0.05; Fig. 5A, 5B), which indicated that DSBs repair was not hindered by treatment with 5-azaC. Subsequently, western blot analysis demonstrated that 5-azaC pretreatment did not inhibit the expression of the non-homologous end-joining (NHEJ) regulators (*DNA-PK, Phospho-DNA-PK (Ser2056), XRCC4*, and *XLF*) and homologous recombination (HR) regulators (*RPA2* and *RAD51*) over time in CNE2 and SUNE1 cells after IR (Fig. 5C). Taken together, these data indicate that 5-azaC enhances radiation-induced apoptosis; however, this was not associated with cell cycle progression and the modulation of the expression of DSBs repair regulators in CNE2 and SUNE1 cells.

### 5-AzaC induces demethylation and the re-expression of epigenetically silenced genes

**Figure 6:**
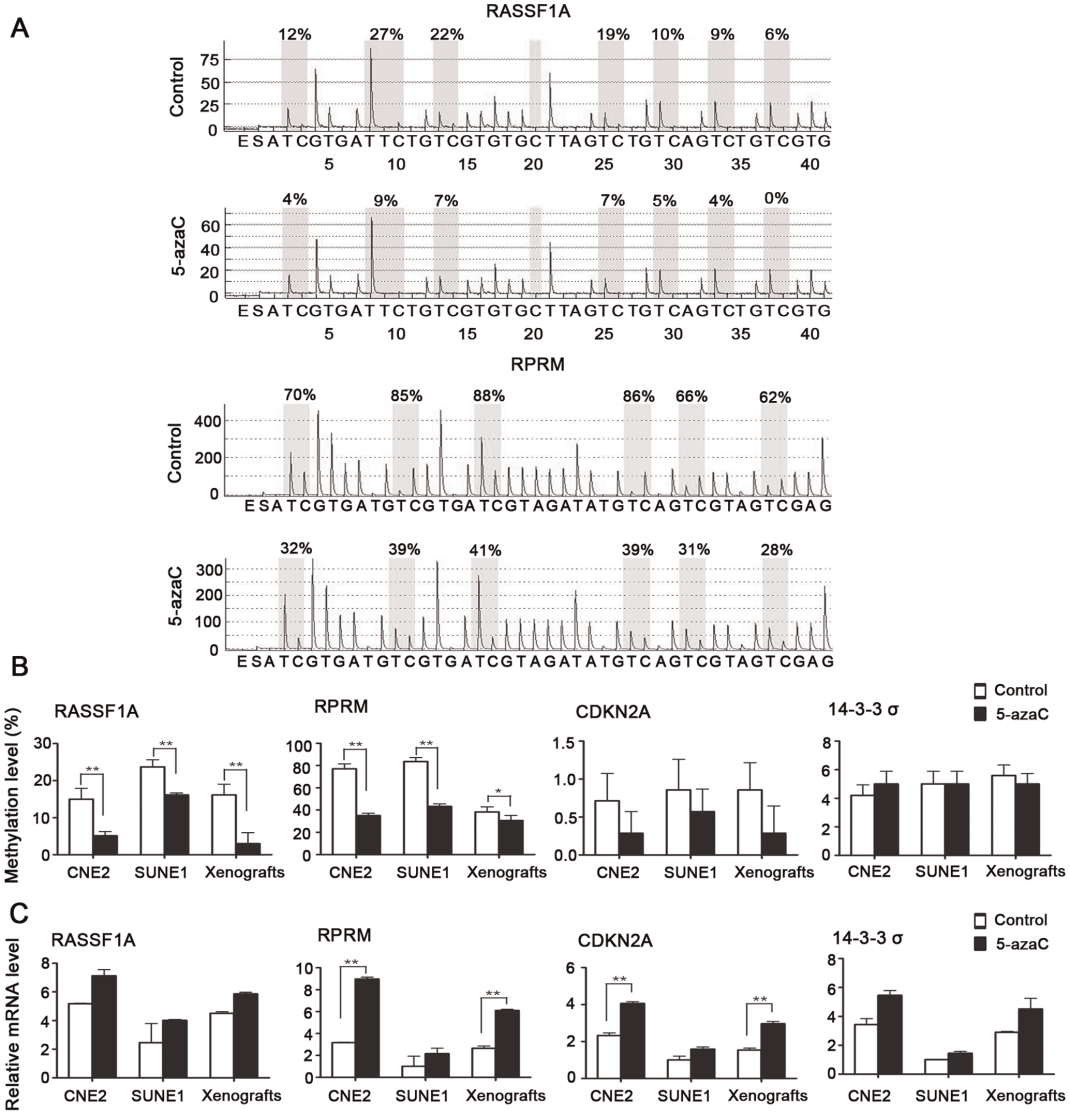
Effect of 5-azaC on the DNA methylation and expression of representative tumor suppressor genes that are hypermethylated and silenced in NPC. SUNE1 and CNE2 cells and mice bearing CNE2 tumor xenografts were treated with or without 5-azaC. (A) DNA methylation pyrograms for *RASSF1A* and *RPRM* in CNE2 cells. (B) Mean levels of DNA methylation for *RASSF1A, RPRM, CDKN2A* and *14-3-3σ* (Student’s *t*-test, *p<0.05, **p<0.01). (C) Real-time PCR analyses of *RASSF1A, RPRM, CDKN2A* and *14-3-3σ* (Student’s *t*-test, **P<0.01).

5-AzaC changes genome-wide methylation status. To further characterize the relevance between gene methylation changes and radiosensitivity, we evaluated the differential methylation and expression status of *RASSF1A, RPRM, CDKN2A*, and *14-3-3σ* both *in vitro* and *in vivo* in NPC by pyrosequencing and real-time RT-PCR. Pyrosequencing revealed that *RASSF1A and RPRM* exhibited hypermethylation at the promoter region, whereas *CDKN2A* and *14-3-3σ* showed hypomethylation at the promoter in CNE2 and SUNE1 cells and in the CNE2 xenografts (Fig. 6A, 6B).

Following 5-azaC treatment, the methylation levels of *RASSF1A* (15.00%±7.75% vs. 5.14%±2.81%, 23.7%±5.05% vs. 16.14%±1.35%, 16.14%±7.75% vs. 3.00%±7.75%, *p*<0.01) and *RPRM* (77.00%±11.10% vs. 35.00%±5.33%, 83.67%±8.69% vs. 43.33%±5.54%, 38.33%±11.10% vs. 30.66%±11.10%, *p*≤0.05) were significantly reduced in both CNE2 and SUNE1 cells and in the CNE2 xenografts, compared to their respective controls (Fig. 6A, 6B). Subsequently, the detection of mRNA expression demonstrated that 5-azaC treatment resulted in an obvious increase in the expression of *RASSF1A, RPRM*, *CDKN2A*, and *14-3-3σ* in both CNE2 and SUNE1 cells and in the CNE2 xenografts. In particular, the increases in the expression of *RPRM* (3-fold upregulation, *p*<0.01; Fig. 6C) and *CDKN2A* (2-fold upregulation, *p*<0.01; Fig. 6C) observed in CNE2 cells and the CNE2 xenografts were significant. Taken together, these results from both *in vitro* and *in vivo* experiments demonstrate that 5-azaC changes methylation levels and restores the expression of mRNA in key genes involved in DNA repair in NPC cell lines.

## Discussion

5-AzaC, as a nucleotide analog, is known to inhibit DNA methyltransferases (DNMTs), which results in DNA hypomethylation and the re-expression of epigenetically silenced genes [18]. In the present study, we evaluated the optimal treatment schedule for the cytotoxicity of 5-azaC *in vitro* and *in vivo* and used minimally toxic drug concentrations combined with radiotherapy for further investigations. Our findings agree with previous studies that showed that concentrations of 1 μmol/L, as used in our current study, did not induce substantial cytotoxicity in colorectal carcinoma or bladder cancer cell lines [6], [32]. Our *in vitro* experiments showed that the clonogenic ability of CNE2 and SUNE1 cells was suppressed by IR alone, and further suppressed by the combination of 5-azaC and IR. In addition, 5-azaC also potentiated X-ray antitumor activity *in vivo*. Our observations confirmed prior reports that this combination results in greater degrees of tumor regression in human cancer cell lines [4], [5], [6].

Based on the potent radiosensitizing activity, further investigations of the mechanism by which 5-azaC mediates its radiosensitizing effect were performed in NPC cells. In general, the ability of the cell to undergo apoptosis is one of the most important mechanisms of radiosensitivity [33]. In this study, nontoxic concentrations of 5-azaC were unable to trigger apoptosis in nonirradiated CNE2 or SUNE1 cells. However, a dramatic increase in apoptosis was induced by 5-azaC during irradiation; consequently, a strong, synergistic, cytotoxic effect is achieved. The findings were in line with those reported in other studies. For example, Hong et al. showed that the radiosensitivity of gastric cancer cells to a demethylating agent was dependent on an increasing rate of apoptosis [4]. Juergen et al. found that apoptosis was strongly increased after combined 4 Gy/5-azaC treatment was administered to head and neck squamous carcinoma cells [34]. These findings suggest that the switching on and off of apoptosis may be the critical event during radiosensitivization.

Cell cycle arrest is one of most common causes of a radiosensitizing effect [5]. Pretreatment with 5-azaC did not affect the cell cycle in both cell lines in our experiment. This result is consistent with findings from colorectal carcinoma, lung cancer, and glioblastoma cell lines [6], [9]. However, breast carcinoma and hepatic cancer cell lines have been shown to undergo G2/M phase arrest after treatment with demethylating agents [35], [36]. This discrepancy may be due to the use of different cell lines or differing concentrations of demethylating agents.

NHEJ and HR are the major repair pathways for DNA DSBs in mammalian species [37]. γH2AX is important for sensing ionizing radiation-induced DSBs [38]. In this study, the number of irradiated cells with γH2AX-positive foci and the expression levels of DNA repair-related proteins (NHEJ or HR) were not significantly inhibited by 5-azaC. Hak et al. [9] found an inverse association between 5-azaC treatment and DSBs repair in lung cancer or glioblastoma cells, whereas Harlinde et al. [39], in accordance with our current findings, found no such association in head and neck squamous carcinoma cells. This discrepancy may result from the use of different cells lines, differences in the demethylating agents used and/or radiation treatment schedules.

5-AzaC can induce genome-wide DNA hypomethylation and alter the expression of a variety of genes [18]. According to methylation status analysis, *RASSF1A* and *RPRM* exhibited promoter hypermethylation, which was consistent with the findings of previous studies [11], [12]. In our experiment, the hypomethylated promoters were detected in *CDKN2A* and *14-3-3σ*, while the promoters were reported hypermethylated in other studies [11], [13]. The difference in measured CpG sites may be responsible for the discrepancy. It was noteworthy that non-cytotoxic concentrations of 5-azaC significantly reduced methylation levels and upregulated the mRNA expression of *RPRM*. A recent report indicated that *RPRM* is a tumor suppressor, and the downregulation of the *RPRM* transcript is associated with promoter methylation. *RPRM* re-expression can activate the downstream effector caspase 3 in apoptotic pathways [16]. In our study, we also observed the re-expression of the tumor suppressor *CDKN2A* without significant changes in methylation. The possible reasons may be the upstream components that are epigenetically silenced in *CDKN2A* signaling network are activated after 5-azaC administration, and their re-expression leads to the upregulation of *CDKN2A* expression either directly or indirectly, a study has found [40]. It has recently been shown that *CDKN2A* is not only a cell cycle checkpoint protein, but is also a master regulator of gene expression in the p16INK4a-cyclinD/cdk4-pRb-E2F1 regulating cell proliferation pathway [41], [42]. E2F1 is an important downstream effector that mediates apoptosis in cancer cells [43]. *CDKN2A* re-expression upregulates E2F1 expression and, subsequently, the induction of apoptotic cell death [44]. Therefore, a possible explanation for radiation-induced apoptosis is that apoptotic pathways in cancer cells are activated by ionizing radiation, and the re-expression of apoptosis-associated genes after 5-azaC treatment, which were previously silenced by epigenetic inactivation, further increases the ability of tumor cells to undergo apoptosis.

In summary, our experiments indicate that 5-azaC potentiate antitumor radiotherapy activity and resulted in greater levels of tumor regression than radiotherapy alone both *in vitro* and *in vivo* in NPC cells. Possible mechanisms of radiosensitization may increase the ability of tumor cells to undergo apoptosis after changes in DNA methylation status, which enables the re-expression of tumor suppressor genes. Our findings suggest that the clinical use of epigenetic modulators may be a promising approach to enhance radiosensitivity during the treatment of NPC.

## Supporting Information

Figure S1 **In vivo drug tolerability study.** (A) Mice bearing CNE2 tumor xenografts were randomized into four groups; each group contained 4 mice. The mice were treated using the following schedule for three weeks: Group 1 received intraperitoneal (*i.p*.) injection of 100 μl of PBS twice weekly. Group 2, group 3, and group 4 received *i.p*. injection of 2 mg/kg, 4 mg/kg, or 8 mg/kg 5-azaC twice weekly, respectively. (A, B) Images of the tumor bearing mice (A) and excised tumors (B). (C, upper part) The animals were treated as shown in and euthanized on day 28 of treatment. (C, lower part) Body weight of the mice during the treatment period (one-way ANOVA, *p*>0.05). (D) The average tumor volume (mm^3^) growth curves for each group are shown (Student’s *t*-test, *p*<0.05). (E) Average weight of the excised tumors (g) at the end of the study (Student’s *t*-test,*P<0.05). A dose of 4 mg/kg 5-azaC did not significantly affect the tumor volume or mouse body weight.
(TIF)
