# Supplementary figures and images for: 5-Azacytidine Enhances the Radiosensitivity of CNE2 and SUNE1 Cells In Vitro and In Vivo Possibly by Altering DNA Methylation
Source: PLoS One. 2014 Apr 1;9(4):e93273. doi: 10.1371/journal.pone.0093273 (PMC3972231; doi:10.1371/journal.pone.0093273)

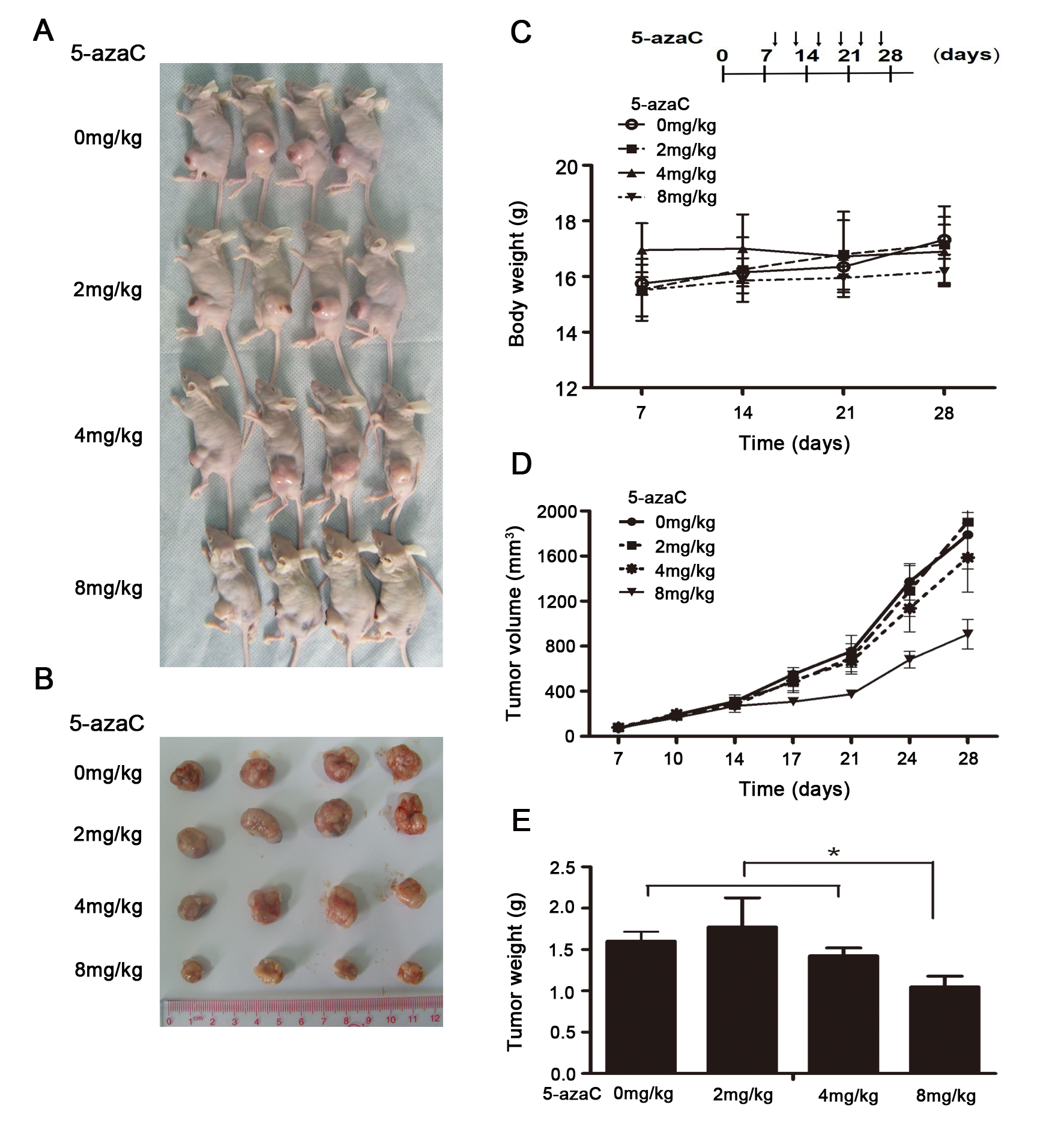

Supplement: Figure S1 — In vivo drug tolerability study. (A) Mice bearing CNE2 tumor xenografts were randomized into four groups; each group contained 4 mice. The mice were treated using the following schedule for three weeks: Group 1 received intraperitoneal (i.p.) injection of 100 μl of PBS twice weekly. Group 2, group 3, and group 4 received i.p. injection of 2 mg/kg, 4 mg/kg, or 8 mg/kg 5-azaC twice weekly, respectively. (A, B) Images of the tumor bearing mice (A) and excised tumors (B). (C, upper part) The animals were treated as shown in and euthanized on day 28 of treatment. (C, lower part) Body weight of the mice during the treatment period (one-way ANOVA, p>0.05). (D) The average tumor volume (mm3) growth curves for each group are shown (Student’s t-test, p<0.05). (E) Average weight of the excised tumors (g) at the end of the study (Student’s t-test,*P<0.05). A dose of 4 mg/kg 5-azaC did not significantly affect the tumor volume or mouse body weight. (TIF) [file pone.0093273.s001.tif]
